# Inferior Vena Cava Filters: A Clinical Review and Future Perspectives

**DOI:** 10.3390/jcm13061761

**Published:** 2024-03-19

**Authors:** Raffaella Benedetti, Simone Marino, Flavio Tangianu, Davide Imberti

**Affiliations:** 1 Haemostasis and Thrombosis Center, Department of Internal Medicine, Hospital of Piacenza, 29121 Piacenza, Italy; r.benedetti@ausl.pc.it (R.B.); d.imberti@ausl.pc.it (D.I.); 2 Department of Internal Medicine, Ospedale di Circolo e Fondazione Macchi, 21100 Varese, Italy; flavio.tangianu@asst-settelaghi.it

**Keywords:** caval filter, pulmonary embolism, deep vein thrombosis, venous thromboembolism

## Abstract

Anticoagulation represents the first line treatment for venous thromboembolism (VTE). However, inferior vena cava (IVC) filter insertion can be considered as a possible therapeutic strategy when anticoagulant therapy is contraindicated, to avoid embolization from the lower limbs to pulmonary circulation. Other possible indications are debated among experts. Both permanent and retrievable caval filters are available in clinical practice. Retrievable filters can be removed when no longer necessary, as their use may be indicated only for a limited amount of time. Moreover, caval filter insertion is not devoid of possible complications, particularly in cases of permanent or long-dwelling filters. A multidisciplinary approach is recommended to review the appropriateness of caval filter use and to define the best timing for retrieval.

## 1. Introduction

Anticoagulation with unfractioned heparin (UFH), low-molecular-weight heparin (LMWH), fondaparinux, vitamin K antagonists (VKA) or direct oral anticoagulants (DOACs) represents the first-line treatment for venous thromboembolism (VTE) [1]. However, when anticoagulant therapy is contraindicated, alternative therapeutic strategies must be considered. In this context, caval filter placement in the inferior vena cava (IVC) represents a good option to avoid embolization from the lower limbs to pulmonary circulation [1].

From a historical point of view, in the second half of the 19th century, Trousseau wrote: “… peut-être que le médecin devrait-il… essayer de mettre une barrière entre le caillot et les portions plus larges du systeme veineux” (“the physician should try to put a barrier between the clot and the larger part of the venous system”) [2].

The first percutaneous IVC filter, the Greenfield filter, was created in 1973 [3]. Since then, caval filter insertion has become the most common alternative VTE treatment in patients with contraindications to anticoagulation. In fact, from 1979 to 2010, their use gradually spread, despite the lack of proven benefits in terms of mortality reduction [4].

In 2010, the Food and Drugs Administration (FDA) issued a warning [5] regarding the possible complications of IVC filter placement, recommending their removal as soon as they are no longer necessary. Since then, many scientific societies’ guidelines have restricted their indications and their use slowly decreased [6,7].

## 2. Types of Caval Filters

All filters are designed to maximize the chance of trapping a thrombus, allowing, at the same time, a normal blood flow through the IVC. They can be classified as permanent (non-retrievable) or optional (retrievable) filters (Table 1).

The first filters introduced in clinical practice were permanent ones, indicated in patients with VTE in the presence of an absolute and prolonged contraindication to anticoagulant therapy [8,9]. However, their use is not devoid of possible long-term complications, such as filter occlusion or recurrence of thrombosis [10,11,12]. Therefore, seeing as the use of IVC filters may be indicated for only a limited amount of time, in the 1990s, retrievable filters were developed [13].

Indeed, retrievable or optional filters are designed to be removed as soon as either the contraindication to anticoagulant therapy or the risk of pulmonary embolism (PE) are solved, but they can also remain in the vena cava permanently, if needed. These filters are removed through an endovascular percutaneous procedure, requiring a device-specific kit to retrieve the filter. 

## 3. Indications to Caval Filter Placement

The main indication to IVC filter placement is a VTE event in the lower limbs in the presence of an absolute contraindication to anticoagulation [1,14,15,16,17,18]. Even though this indication is supported by most scientific societies’ guidelines, no benefit in terms of mortality reduction is demonstrated in patients treated with caval filter insertion, compared to those untreated. On the other hand, it would be unethical to perform a randomized controlled trial (RCT) with patients not eligible for anticoagulant therapy without offering them the only alternative recommended treatment.

Other possible indications to IVC filter placement have been proposed (Table 2). However, solid evidence is lacking in this setting and guideline recommendations, mostly based on expert consensus, are rather inconclusive or conflicting.

Among the few available studies concentrating on the effectiveness of caval filters, most have a small sample size, are observational and only rarely are they prospective, randomized studies. Therefore, they offer a low quality of evidence. Two prospective, randomized controlled trials focusing on the effectiveness of caval filters in addition to anticoagulation have been published.

In the PREPIC study [11], 400 patients with proximal DVT at high risk of embolization were randomized to treatment with permanent caval filter in addition to anticoagulant therapy or anticoagulation alone. The study’s primary endpoint was the incidence of pulmonary embolism at day 12. In patients who underwent caval filter placement in addition to anticoagulant therapy, the researchers observed a lower incidence of PE at day 12 (1.1% vs. 4.8%; OR 0.22, 95% CI, 0.05–0.90; *p* = 0.03). Nevertheless, at 2 years, this benefit was lost, both in terms of risk of embolization (3.4% vs. 6.3%; OR 0.50, 95% CI, 0.19–1.33; *p* = 0.16) and in terms of mortality (21.6% vs. 20.1%; OR 1.10, 95% CI, 0.72–1.70; *p* = 0.65). Furthermore, the results show a statistically significant increase in the risk of DVT recurrence (20.8% vs. 11.6%; OR 1.87, 95% CI, 1.10–3.20; *p* = 0.02) for patients in the caval filter group.

During the 8 years follow-up period, patients treated with caval filter placement and anticoagulation showed a lower incidence of PE, compared with those treated with anticoagulation alone (6.2% vs. 15.1%; *p* = 0.008) [19].

A second randomized controlled study, the PREPIC 2 study [20], was conducted after the introduction of retrievable filters. This study focused on 399 patients with PE and symptomatic DVT at high severity risk (age > 75 years, active cancer, respiratory failure, ischemic stroke with paralysis of a lower limb in the last 6 months, bilateral DVT, iliac vein or IVC thrombosis, presence of at least one sign of ventricular dysfunction or myocardial injury: right ventricle dilation, pulmonary hypertension, high levels of BNP, NT-proBNP, Troponine T or Troponine I). All patients were treated with anticoagulant therapy for at least 6 months; the study group received an additional treatment with retrievable caval filter insertion, with planned removal at 3 months. Filters were effectively retrieved in 153 out of 193 patients. The primary efficacy endpoint of the study was the recurrence of fatal or symptomatic non-fatal PE at 3 months, while the secondary efficacy endpoints were recurrence of fatal and symptomatic non-fatal PE at 6 months or a new episode or recurrence of DVT at 3 and 6 months.

No significant differences on efficacy endpoints were found at 3 and 6 months between the two groups, but a trend towards an increased risk of recurrent PE and mortality was noted in patients treated with caval filter placement. The authors concluded that these results did not support the use of caval filters in patients who could be treated with anticoagulation.

Other studies with smaller sample sizes were conducted to determine the possible benefits of the use of caval filters in selected populations. For instance, a small study that included patients with recurrent VTE despite anticoagulant therapy [21] demonstrated that caval filter placement reduced all-cause mortality (2.15 vs. 25.3%; *p* = 0.02) and mortality due to pulmonary embolism (2.1% vs. 17.6%; *p* = 0.08) at 3 months in patients with recurrent PE. The same benefit was not shown in patients with recurrent DVT, both for all-cause mortality (17.7% vs. 12.2%; *p* = 0.56), and mortality due to PE (0% vs. 0%; *p* = n.s.).

In rare cases, patients with massive iliac-femoral thrombosis at high risk of limb gangrene are treated with thrombolytic therapy, which is a possible cause of embolization. In such cases, caval filter placement has been proposed as a possible strategy to prevent PE [22,23]. However, there is no strong evidence in favor of the use of caval filters, both permanent and retrievable, during thrombolysis [24].

For a long time, the finding of a free-floating venous thrombus in patients with proximal DVT was considered a high-risk factor for PE, regardless of adequate anticoagulation, and therefore, the use of caval filters was proposed in this situation [25,26,27]. However, the actual relevance of this finding as a risk factor has been reconsidered on the basis of the results of a few well-conducted prospective clinical trials and, moreover, the effectiveness of caval filters in preventing PE in this setting has not been demonstrated [28].

Recently, the PRESERVE Trial [29] evaluated the safety and effectiveness of caval filter use. In total, 1429 patients who required caval filter placement due to a contraindication to anticoagulation or therapeutic failure were enrolled; each patient was periodically evaluated after caval filter insertion with a clinical and radiological follow-up.

The primary safety endpoint was freedom from serious adverse events in the periprocedural period and during the 12 months following filter placement. The primary effectiveness endpoints included procedural success rate and absence of clinically significant PE at 12 months (or 1 month after retrieval). Caval filters were placed in 1421 of the enrolled patients. Most of them (71.3%, 1019 patients) had acute or chronic VTE, while only 8.9% of patients (n = 127) had no current or prior VTE. A 6.5% incidence of VTE was estimated in the 12 months following caval filter placement, including 80 cases of DVT (5.2%), 23 cases of PE (1.6%) and 15 cases of filter occlusion (1.1%). No PE was reported in patients who underwent prophylactic filter placement. Both the primary safety and effectiveness endpoints were met, with a procedural success rate of 98% (95% CI, 97.2–98.7%) and a 98.3% rate of freedom from significant PE (95% CI, 97.2–99.1%) at 12 months for in-site filters or at 1 month after retrieval. This study confirms the general safety of IVC filters, with a relatively low rate of adverse events. However, the lack of a control group in this study and the possible impact of concomitant anticoagulant treatment before, during or after IVC filter insertion complicate the evaluation of possible benefits associated with caval filter use.

## 4. Use of Caval Filters in Special Populations

### 4.1. Poor Cardiopulmonary Reserve and Hemodynamic Instability

Untreated acute PE is burdened with a high mortality rate. In patients with PE and a contraindication to anticoagulant therapy, IVC filter placement represents a possible option to avoid recurrent embolization and clinical deterioration. Despite the paucity of evidence, recent guidelines [30] support this strategy, suggesting an individualized approach to IVC filter placement in this context, which should take into consideration factors such as the patient’s individual thromboembolic risk, cardiopulmonary reserve and overall hemodynamic stability.

Furthermore, the Society of Interventional Radiology recently recommended IVC filter placement in selected patients with acute PE treated with advanced therapies, such as thrombolysis, thrombectomy or embolectomy [30]. In fact, a few observational studies [31,32] found a significant reduction in in-hospital mortality (7.6% vs. 18%, RR 0.43, 95% CI, 0.39–0.47) in unstable patients with acute PE treated with thrombolytic therapy and IVC filter placement, compared with those without an IVC filter. A study by Stein et al. [33] demonstrated that in unstable patients with acute PE (e.g., shock or need of ventilatory support) treated with thrombolytic therapy or embolectomy, all-cause mortality was lower in patients with a caval filter, compared to those without it (21% vs. 48%, RR 0.44, 95% CI, 0.33–0.59). On the other hand, another study [34] found no significant difference in terms of mortality in hemodynamically unstable patients with PE treated with thrombolysis associated with IVC filter placement (HR 0.86, 95% CI, 0.6–1.21).

Therefore, IVC filter insertion should be considered in selected patients with a contraindication to anticoagulant therapy when the risk of clinical deterioration due to acute or recurrent PE outweighs the risk associated with caval filter placement.

### 4.2. Pregnancy and VTE

With an incidence of about 1 case per 1000 pregnancies, VTE is not a rare occurrence during pregnancy and puerperium [35]. VTE risk is 5 times higher during pregnancy than in non-pregnant women and the risk increases even more in the post-partum period [36]. On the other hand, anticoagulant treatment might expose the patient to a high bleeding risk, particularly in peripartum. Therefore, it may be necessary to temporarily stop anticoagulation to avoid complications. In this situation, the use of a retrievable caval filter is tempting. As a general principle, IVC filters have the same indications in pregnant women as in the general population and should be considered in cases of contraindication, therapy failure or significant complications to anticoagulation (e.g., bleeding) [36]. However, pregnant women have been excluded from randomized studies on caval filters (including the PREPIC and PREPIC 2 studies), and all evidence regarding the safety and effectiveness of caval filter use during pregnancy is derived mainly from case reports [37]. Moreover, it should be noted that pregnant patients may present a higher risk of filter-related complications, such as filter fracture, tilting or migration, due to physiologic changes during pregnancy (e.g., increased vena cava size and course deviation) [38].

In conclusion, due to the lack of data on the effectiveness and safety of IVC filters in this population, their use should be limited to selected cases of pregnant women considered at high VTE risk (e.g., recurrent VTE despite anticoagulation) [39,40]. Decisions regarding IVC filter placement in pregnant women should be carefully considered, weighing possible risks and benefits and treatment choices should be evaluated by a multidisciplinary team.

### 4.3. VTE and Cancer

Oncologic patients have a higher risk of VTE events and an increased risk of morbidity and mortality related to VTE compared to the general population. Furthermore, as these patients also present an increased bleeding risk, the use of caval filters may represent an obvious solution. A small prospective randomized study on 64 oncologic patients with VTE did not show any benefit associated with caval filter use [41]. Another study based on a database of 14,000 patients, 19.6% of whom were treated with caval filter placement, showed that the use of caval filters did not reduce the risk of mortality and recurrence of PE, but was associated with an increased risk of DVT at 180 days [42]. A recent study [43] evaluated the association of IVC filter placement with the prevention of PE and subsequent DVT in a total of 88,585 patients with cancer and a new diagnosis of DVT. Of these, 33,740 (38.1%) underwent caval filter insertion and 4492 patients (5.1%) developed PE after the initial diagnosis. Patients who underwent IVC filter placement had a lower risk of developing PE (HR 0.69, 95% CI, 0.64–0.75), compared to those without a caval filter. Moreover, IVC filter use did not appear to increase the risk of further DVT, when accounting for differences in anticoagulation and individual risk factors.

The 2019 American Society of Clinical Oncology (ASCO) Clinical Practice Guideline Update on VTE prophylaxis and treatment recommended IVC filter insertion only in cases of an absolute contraindication to anticoagulant therapy in patients with life-threatening VTE in the acute phase (within 4 weeks). Additionally, the panel of experts suggested IVC filter placement in addition to anticoagulation in patients with progression of VTE despite optimal anticoagulant therapy [44].

The role of IVC filter placement in cancer patients remains uncertain because of the scarcity of data regarding this population and of the possible complications associated with the procedure. Caval filter placement should, therefore, be considered only in selected patients with cancer after taking into consideration individual risk factors.

### 4.4. VTE Prophylaxis

The prophylactic use of IVC filters can be considered in patients at high risk of VTE, mainly in the context of polytrauma and in surgical patients who cannot receive a pharmacologic thromboprophylaxis [25,45,46].

In cases of major trauma, reduced mobility, endothelial injury and hypercoagulability are responsible for the patient’s high thromboembolic risk: pulmonary embolism is the cause of death in 20% of cases in this population [47,48]. On the other hand, pharmacologic anticoagulant prophylaxis significantly increases the patients’ bleeding risk. In this context, the use of an IVC filter for VTE prophylaxis in trauma patients remains controversial, as there is a lack of solid evidence supporting this practice.

Two retrospective studies showed a reduced incidence of symptomatic and fatal PE in trauma patients treated with prophylactic vena cava filters [49,50]; however, these findings were not confirmed by subsequent studies, which, in contrast, demonstrated an increased incidence of DVT in these patients [51,52].

In a recently published RCT [45], caval filter placement in the first 72 h from presentation did not confer any protection in terms of all-cause mortality and risk of PE at 90 days in patients with severe polytrauma and a contraindication to pharmacologic prophylaxis (HR 0.99, 95% CI, 0.51–1.94, *p* = 0.98). However, no pulmonary thromboembolic events were reported in patients with prophylactic caval filter who survived for more than 7 days after trauma and had a prolonged contraindication to anticoagulation. On the other hand, PE occurred in 14.7% of patients without caval filter. Therefore, it is possible to speculate that while not all major trauma patients would benefit from caval filter implantation, this option could be reserved for patients with a prolonged contraindication to pharmacologic prophylaxis.

Several studies showed the effectiveness of caval filter placement in preventing pulmonary embolism in surgical patients at high thromboembolic risk [53,54,55]. However, none of these studies included a control group and all of them provided a limited follow-up period. Moreover, as several safe, effective and handy pharmacologic options are available for thromboprophylaxis (LMWH, fondaparinux, DOACs), the use of optional caval filters is nowadays mostly limited to only a few, highly selected, cases.

Even though bariatric surgery is associated with a very high thromboembolic risk, no benefit has been demonstrated in the use of prophylactic caval filters, even in severely obese patients with a BMI > 55 kg/m^2^ [56]. A meta-analysis of 18 studies showed that prophylactic caval filter placement in this context was beneficial only in patients presenting multiple thromboembolic risk factors, with a significant reduction in mortality related to pulmonary embolism [57].

## 5. Caval Filters Complications

Complications associated with caval filters can occur immediately after filter insertion but, for the most part, they arise after the first month and are related to a prolonged permanence of the filter.

Early complications can be due to procedural complications, such as bleeding or infection at the site of venipuncture, development of arteriovenous fistulas, accidental arterial puncture and post-procedural hematoma or thrombosis. Other early complications are filter-related, such as malposition, incomplete expansion, caval penetration or guidewire entrapment.

Delayed complications, such as deep vein thrombosis, filter occlusion, migration and fracture or caval rupture and thrombosis, can be accidentally diagnosed during imaging studies obtained for other reasons or during a planned caval filter retrieval. They rarely happen in the first month after insertion [58].

DVT is one of the most common delayed complications of caval filter insertion. The risk of DVT depends on the type of filter (4–18%) and increases with a prolonged filter permanence [59]. In the PREPIC study, the incidence of recurrent DTV after 2 years of follow-up was significantly higher in patients treated with caval filter implantation, compared to those without filters (20.8% vs. 11.6%, *p* = 0.02) [11]. This difference persisted even after 8 years of follow-up [19], with a 34.1% incidence of recurrent DVT in patients with an implanted caval filter versus a 27.3% incidence in the group without caval filters (*p* = 0.042). A retrospective study of more than 80,000 patients hospitalized for DVT showed that subjects treated with caval filter insertion had a higher incidence of recurrent DVT at 1 year compared to those without caval filters (5.3% vs. 3.7%) [60].

Caval filter occlusion can result from the filter’s thrombogenicity, progression of a distal thrombosis towards the vena cava or due to a captured embolus. Filter occlusion can cause significant side effects, such as reduced protection against PE, filter migration and post-thrombotic syndrome. In the past decades, the incidence of filter occlusion varied between 6% and 30% [10]. However, new-generation filters seem to be associated with a lower inherent thrombogenicity, as demonstrated by a recent meta-analysis, which reported a 2.8% incidence of filter thrombosis [59].

Filter migration to the heart is a potentially life-threatening delayed complication [61], but in most cases, migration is less extensive and is not associated with significant morbidity. A meta-analysis reported a 1.3% rate of events, with 90% of cases taking place after more than 30 days since filter placement [59].

Vena cava perforation is an occasional radiologic finding, usually completely asymptomatic and devoid of any clinical significance; it usually happens with long dwelling vena cava filters, tilted more than 15 degrees. Caval perforation is a common event, representing up to 20% of all filter-related complications [59].

## 6. Optional Caval Filters Removal

As the risk of complications depends on the filter’s permanence time, optional filters should be removed as soon as the patient is no longer at risk of pulmonary embolism. Nevertheless, only one third of optional filters is removed when no longer necessary [59].

On the other hand, certain conditions might preclude caval filter retrieval, such as advanced age, impaired cardio-pulmonary function, comorbidities (chronic kidney disease, advanced cancer, neurologic conditions), permanent contraindications to anticoagulant therapy, recurrent VTE during appropriate anticoagulation and patients’ refusal [62,63]. Such potential situations must be considered before filter implantation and should be taken into account when choosing the type of filter (permanent or optional), as their presence favors the choice of a permanent filter.

Furthermore, filter retrieval might become technically unfeasible, for instance, in the presence of a large thrombus (over 30% of the filter’s volume) or in cases of wall penetration. Caval filter tilting does not preclude retrieval but can be associated with a high failure rate of the procedure [64].

It has been demonstrated that in trauma patients there is a higher risk of complications during retrieval after 6 months from insertion [65].

An Italian observational study showed that retrieval safety depends on its timing and, in particular, its success rate is higher when attempted within 3 months after implantation [61].

Considering that the longer the filter dwelling time, the higher is the risk of complications, while, at the same time, the earlier the retrieval attempt, the greater the success rate, it is of extreme importance to remove optional filters within the optimal time frame.

The lack of a planned follow-up for patients is the main reason why optional filters are not retrieved [58]. It is, therefore, necessary to encourage any program aimed at raising awareness on the importance of a systematic follow-up for these patients [66,67,68,69] to improve the rate of filter retrieval. Thus, different strategies and organizational models have been proposed. Firstly, it is important that the patient is aware and informed of the temporary nature of the device. Possible strategies include the creation of teams dedicated to VTE patients, in charge of reviewing the indication of caval filter placement, choosing the type of filter, and planning its removal. Other solutions are the creation of hospital services dedicated to filter removal, the early planning of the filter’s reevaluation date, at the moment of its placement, and the use of filter “registers”, which are associated with a 15.5–31% increase in filter retrieval rate. Finally, another option is the use of computerized systems sending notifications to both patient and physician, reminding them of the filter’s planned retrieval date. Each strategy has costs and possible advantages, which should be carefully evaluated, depending on local resources. Whatever the chosen strategy, a systematic approach to the patient’s follow-up is essential [69].

## 7. Guidelines

All main international guidelines agree that caval filter placement is indicated in cases of venous thromboembolism associated with an absolute contraindication to anticoagulation. The American College of Chest Physicians (ACCP) recommends caval filter placement in patients with acute deep vein thrombosis only in cases of contraindications to anticoagulant therapy. When anticoagulation is feasible, the ACCP guidelines recommend against caval filter insertion (strong recommendation, moderate quality of evidence) [70]. In 2020, the Society of Interventional Radiology recommended against routine IVC filter placement in patients treated with anticoagulant therapy as it may increase the risk of DVT, without any relevant benefit in terms of mortality reduction, compared to patients treated with anticoagulation alone [30].

The European Society of Cardiology, in its guidelines, recommends the use of caval filters in patients with acute PE and an absolute contraindication to anticoagulant therapy. Another suggested indication is the progression of pulmonary embolism despite appropriate anticoagulant therapy [1].

According to the NICE guidelines, caval filter placement is also indicated in patients with recurrent VTE despite adequate anticoagulation or in the context of clinical trials [71].

Guidelines have different recommendations on the use caval filters in polytrauma patients for VTE prophylaxis. Some suggest caval filter placement in all high-risk trauma patients, while others recommend it only for high-risk patients with prolonged hypomobility and contraindication to pharmacologic prophylaxis. The 2016 ACCP guidelines recommended against the use of prophylactic caval filters; this indication was not modified in the 2021 update [70].

In a recent document by the Italian Society for Thrombosis and Haemostasis (SISET) regarding various issues related to caval filter use, all the experts involved agreed that vena cava filters used in association with anticoagulant therapy did not significantly reduce mortality after an acute thromboembolic event, even though they could reduce the incidence of PE after acute DVT. No unanimous position was reached regarding the use of vena cava filters to reduce the risk of recurrence in patients with isolated PE. All authors agreed about the necessity of retrieving caval filters as soon as the contraindication to anticoagulant therapy is removed, possibly within 90–120 days after implantation [72].

## 8. Future Perspectives

New convertible devices have been developed to guarantee adequate protection against PE while, at the same time, avoiding possible complications due to prolonged dwell time. These new filters do not need retrieval as they can be converted through interventional radiology procedures to an “open”, non-filtering, configuration when mechanical prophylaxis is no longer necessary. A study with 149 patients demonstrated a high percentage of filter conversion and a low incidence of adverse events [73].

Bioconvertible filters represent another alternative to retrievable filters for patients with a contraindication to anticoagulation and a transient risk of PE. They can automatically convert to an “open” configuration, without the necessity for interventional procedures, thanks to the presence of a bioabsorbable fragment placed at the center of the vascular lumen, holding together the filter’s arms. When this fragment is degraded through hydrolysis, the filter’s arms retract, adhering to the caval wall and then become endothelialized, leaving the vein unfiltered [74]. A recent prospective multicenter trial [75] evaluated the 2-year outcomes associated with bioconvertible IVC filter use. In total, 129 patients with a diagnosis of VTE or who were at risk of developing DVT or PE underwent filter implantation. They were then periodically evaluated to monitor the filter’s status and detect complications through a 2-year clinical follow-up with radiography, computed tomography (CT) or CT venography. Bioconversion was achieved in 95.7% of patients at 6 months and 96.4% at 24 months, without signs of obstruction or thrombosis related to the filter’s retracted arms in its “open” configuration. During the first 12 months, no cases of symptomatic PE were detected, while at 24 months the rate of symptomatic PE was 2.4%. However, two cases of symptomatic caval thrombosis were reported after implantation.

In addition, a new percutaneous and removable IVC filter has been introduced for critically ill patients at high thromboembolic risk or with a recent diagnosis of VTE and a contraindication to anticoagulant therapy [76]. It consists of an IVC filter attached to a triple lumen central venous catheter, which can be inserted via the femoral vein and then easily removed when no longer necessary. Data derived from surgical and trauma patients [77,78] showed a lower incidence of PE and a reduction in mortality associated with this new device. In a recent study [79], 18 critically ill medical patients with a diagnosis of VTE (67% PE and 72.2% DVT) or at high thromboembolic risk presenting a contraindication to anticoagulation underwent catheter placement. The median duration of these catheters was 5 days. No cases of recurrent PE or DVT were detected, but in five cases (29.4%) a clot was found in the filter at the moment of catheter removal.

Bioabsorbable filters have been proposed, which are made with materials engineered to dissolve with time. Studies on animal models showed that filter degradation products did not cause harm to the lungs [80,81].

The use of optional filters medicated with rapamycin and heparin has been proposed to improve filter removal success rates. In vitro and in vivo studies demonstrated that medicated filters could effectively reduce endothelial proliferation, while the anticoagulant effect of heparin in this context was unsatisfactory [82]. More studies are necessary to verify the possible application of these new filters in clinical practice.

Lastly, new procedures have been proposed for caval filter retrieval, in particular in the context of long-dwelling IVC filters, which are notoriously difficult to remove with standard techniques and are at risk of complications (e.g., fracture, migration or thrombosis) [83,84]. For instance, a recent study [85] evaluated the use of the excimer laser sheath for retrieval of embedded IVC filters, a device commonly used for the extraction of pacemaker and defibrillator leads. This procedure was associated with a high success rate (95.2%, 95% CI, 89.9–98.2%, *p* = 0.02) and a low rate (4%, 95% CI, 1.3–9.0, *p* = 0.01) of major complications, defined as any adverse event associated with morbidity or disability or resulting in hospital admission. However, the standard snare technique remains at the moment the first choice for filter retrieval, as “advanced” techniques, such as the laser sheath technique, should be performed only in selected cases and by expert operators [86].

## 9. Conclusions

Caval filter placement is an alternative therapeutic strategy for patients with VTE and a simultaneous permanent or temporary contraindication to anticoagulation. Other possible indications are still debated. Both permanent and retrievable filters are available in clinical practice. The use of permanent filters is often burdened by major complications. On the other hand, the longer the optional filter’s permanence time, the greater the difficulty and the lower the success rate of the retrieval procedures. Therefore, removing caval filters as soon as they are no longer indicated is essential. Thus, every healthcare facility should create a multidisciplinary team comprising, among others, experts in hemostasis and thrombosis, interventional radiologists and vascular surgeons [87]. Such teams should be in charge of reviewing the appropriateness of caval filter use and of planning an adequate follow-up to monitor complications and, when possible, define the best timing for filter retrieval. In the near future, it is likely that further development of absorbable filters will lead to reviewing the current indications on the use of caval filters.

## Figures and Tables

**Table 1:** Commonly used caval filters.

Producer	Name	Type
Boston Scientific	Greenfield	Permanent
B. Braun	VenaTech	Permanent
ALN	ALN vena cava filter	Optional
Bard	Denali	Optional
Cook	Celect	Optional
Cook	Günther Tulip	Optional
Cordis	OPTEASE	Optional
Argon	OptionELITE	Optional
Cook	Bird’sNest	Permanent
Bard	Recovery G2	Optional

**Table 2:** Caval filters indications.

Appropriate Indications
- Acute VTE with temporary contraindication to anticoagulation
Possible indications
- Thrombolysis for iliac-caval thrombosis
- High bleeding risk during peripartum and puerperium
- Prophylaxis in patients at high thromboembolic risk who cannot be anticoagulated
- VTE prophylaxis in surgical/orthopedic patients at high thromboembolic risk
- VTE prophylaxis in bariatric surgery
- VTE recurrence or progression during anticoagulation
- Massive pulmonary embolism treated with thrombectomy or thrombolysis

## Data Availability

Not applicable.
